# Identification of a novel gene fusion in ALT positive osteosarcoma

**DOI:** 10.18632/oncotarget.26029

**Published:** 2018-08-28

**Authors:** Emily Mason-Osann, Anqi Dai, Jess Floro, Ying Jie Lock, Matthew Reiss, Himabindu Gali, Adeline Matschulat, Adam Labadorf, Rachel Litman Flynn

**Affiliations:** ^1^ Departments of Pharmacology and Experimental Therapeutics, and Medicine Cancer Center, Boston University School of Medicine, Boston, MA 02118, USA; ^2^ BU Bioinformatics Hub, Boston University, Boston, MA 02118, USA; ^3^ Department of Biochemistry, Boston University School of Medicine, Boston, MA 02118, USA

**Keywords:** telomeres, alternative lengthening of telomeres

## Abstract

The Alternative Lengthening of Telomeres (ALT) pathway stimulates telomere elongation and prevents cellular senescence in approximately 60% of osteosarcoma. While the precise mechanism underlying activation of the ALT pathway is unclear, mutations in the chromatin remodeling protein ATRX, histone chaperone DAXX, and the histone variant H3.3 correlate with ALT status. ATRX and DAXX facilitate deposition of the histone variant H3.3 within heterochromatic regions suggesting that loss of ATRX, DAXX, and/or H3.3 lead to defects in the stability of telomeric heterochromatin. Genetic mutations in ATRX, DAXX, and H3.3 have been detected in ALT positive cancers, however, a subset of ALT samples show loss of ATRX or DAXX protein expression or localization without evidence of genetic alterations suggesting additional uncharacterized defects in ATRX/DAXX/H3.3 function. Here, using Next Generation Sequencing we identified a novel gene fusion event between DAXX and the kinesin motor protein, KIFC3, leading to the translation of a chimeric DAXX-KIFC3 fusion protein. Moreover, we demonstrate that the fusion of KIFC3 to DAXX causes defects in DAXX function likely promoting ALT activity. These data highlight a potentially unrecognized mechanism of DAXX inactivation in ALT positive osteosarcoma and provide rationale for thorough and comprehensive analyses of ATRX/DAXX/H3.3 proteins in ALT positive cancers.

## INTRODUCTION

Telomere elongation is a requisite for cellular immortality and a hallmark of cancer cells. The majority of cancer cells rely on reactivation of the enzyme telomerase or activation of the Alternative Lengthening of Telomeres pathway (ALT) to promote telomere elongation. The telomerase holoenzyme promotes telomere elongation by using the RNA component TERC as a template for reverse transcription at telomere ends. ALT, however, promotes telomere elongation using homology directed DNA damage repair via break induced replication [1–4]. Although it is unclear exactly how the recombinogenic state of ALT telomeres is established, uncapped or dysfunctional telomeres are believed to be an early event in the process [5, 6].

ALT is a recombination-based mechanism where one telomere uses other chromosomal, or extrachromosomal, telomeric DNA sequences as a template for telomere elongation. ALT was recently described to resemble break-induced replication (BIR), a specific homology directed repair pathway utilized to repair one-sided DNA double-stranded breaks that are often generated following replication fork collapse [4]. Supporting this mechanism, ALT telomeres are characterized by high levels of replication stress and consequently, spontaneous DNA damage [5, 7–9]. Additionally, telomeres in ALT cells are found to be extremely heterogeneous in length, further supporting a role for recombination in ALT. ALT cells are also characterized by a number of unique cellular phenotypes including the formation of ALT associated PML bodies (APBs) [10]. APB are nuclear domains that in addition to the PML protein, contain telomeric DNA and a number of recombination and repair factors hypothesized to mediate ALT activity. APB are unique to cells that rely on ALT for telomere elongation and have been used on clinical specimens to determine ALT status [11, 12].

Genetically, ALT positive cancers frequently harbor inactivating mutations in the gene loci of α-thalassemia/mental retardation syndrome X-linked (ATRX), Death domain associated protein (DAXX), and H3 histone family member 3A (H3F3A/H3.3) [6, 13, 14]. ATRX and DAXX form a complex that is responsible for the deposition of the histone variant H3.3 at heterochromatic regions to ensure transcriptional silencing and heterochromatin integrity [15–19]. Functional inactivation of ATRX, DAXX, or H3.3 lead to defects in heterochromatin formation at highly repetitive loci including pericentric, telomeric, and retroviral DNA repeats. This has led to the hypothesis that defects in heterochromatin maintenance induce telomere dysfunction and contribute to the stimulation of ALT.

Although genetic mutations in ATRX and DAXX are often demonstrated in ALT positive cancers, a fraction of cancers display significant defects in DAXX/ATRX protein expression and/or function that do not stem from genetic mutations [6]. This has raised the possibility that other mechanisms likely contribute to functional inactivation of DAXX/ATRX proteins. Consistent with these studies, our data identify a novel structural rearrangement between the 3′UTR of DAXX and the kinesin family member KIFC3. Moreover, we show that the resultant fusion protein compromises DAXX function and contributes to the ALT phenotype. Our data provide additional support for functional inactivation of the ATRX/DAXX/H3.3 pathway in the absence of genetic mutations in the coding region of ATRX, DAXX, or H3.3 genes.

## RESULTS

### Identification of DAXX fusion transcript in ALT positive osteosarcoma

There is a strong correlation between genetic mutations in ATRX/DAXX/H3.3 and cancers that rely on the ALT pathway for telomere maintenance. However, a number of ALT positive tumors retain wild-type ATRX and DAXX gene loci yet demonstrate defects in ATRX and DAXX protein expression. These data suggest that functional inactivation of the ATRX/DAXX/H3.3 axis may occur at the RNA or protein level. Therefore, we asked whether we could identify defects in ATRX/DAXX/H3.3 using RNA sequencing on a panel of 13 osteosarcoma cell lines. Of these cell lines, 8 (U2OS, SAOS2, CAL72, CAL78, NY, NOS1, G292, and HUO9) have been previously characterized as ALT positive and 5 (HOS, MG63, SJSA1, hFOB1.19, and HUO3N1) have been characterized as ALT negative (Supplementary Figure 1, Supplementary Figure 2A–2B, and Supplementary Figure 3A–3D) [6, 20]. Although ALT status had been analyzed across the panel of cell lines CAL78, NY, and G292 were the only ALT positive cell lines that retained ATRX/DAXX/H3.3 protein expression [6, 20] (Supplementary Figure 1). Following RNA sequencing and western blot analysis we found that CAL78 and NY demonstrated loss of expression of the SWI/SNF related, matrix associated, actin dependent regulator of chromatin, subfamily a like 1 (SMARCAL1) gene and a corresponding loss of full length SMARCAL1 protein expression (Supplementary Figure 1 and Supplementary Figure 3D). Likewise, a recent publication reported SMARCAL1 mutations in ALT positive glioblastomas as well as in the CAL78 cell line [21]. SMARCAL1 is an annealing helicase that functions to remodel chromatin surrounding sites of stalled replication forks and has been demonstrated to function to resolve replication stress at telomeric DNA suggesting that defects in SMARCAL1 function lead to the activation of the ALT pathway [9, 22].

**Figure 1:**
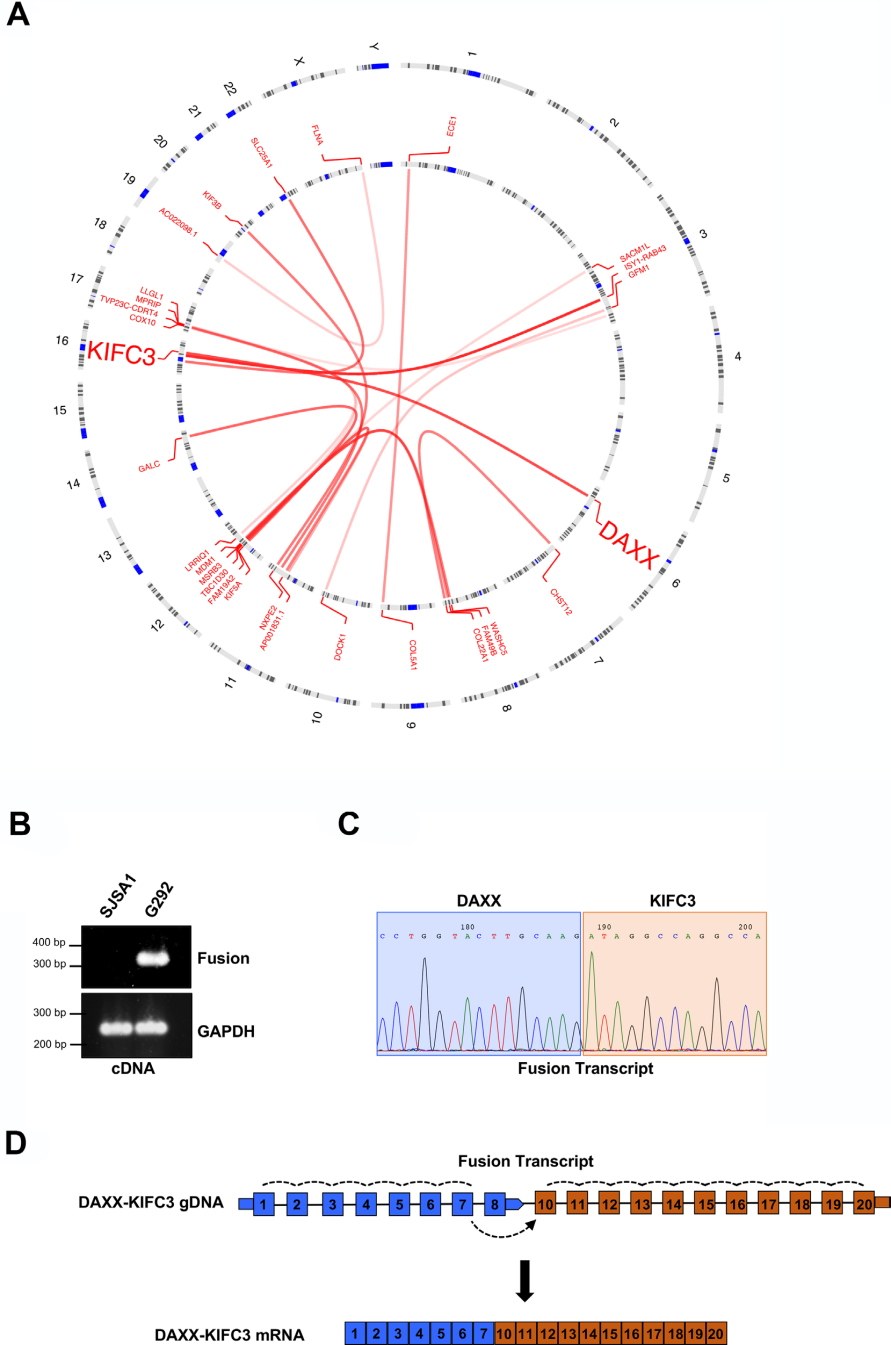
Identification of a DAXX-KIFC3 fusion transcript in an ALT positive osteosarcoma (**A**) Circos plot generated using the OmicCircos package following RNA sequencing of the G292 cell line. Lines represent the predicted interchromosomal fusion transcripts. (**B**) RT-PCR amplification products from SJSA1 and G292 RNA using primers that either flank the predicted transcriptional fusion site or amplify GAPDH as a control. (**C**) Representative chromatogram of the Sanger sequencing results from the RT-PCR product generated from G292 RNA. Sequence shown highlights the region where the fusion event between DAXX and KIFC3 occurs. (**D**) Schematic of the fusion transcript that contains exons 1–7 of DAXX and exons 10–20 of KIFC3. Dotted lines indicate splicing events.

Out of the 8 ALT positive cell lines analyzed, G292 was the only remaining cell line without a characterized defect in the genes currently associated with ALT. Therefore, in addition to analysis of simple gene expression, we also analyzed putative gene fusion events across our 13 cell lines and identified chimeric reads in the ALT positive G292 cell line data that aligned to both the DAXX and the kinesin family member, KIFC3 gene loci (Figure 1A and Supplementary Figure 4). These chimeric reads suggested a transcriptional fusion between DAXX and the kinesin family member, KIFC3. The DAXX-KIFC3 chimeric reads suggested two transcript fusion events, a less abundant event located between position c.33318688 on chromosome 6 and c.57766986 on chromosome 16 and a more highly expressed event between c.33318996 on chromosome 6 and c.57766986 on chromosome 16 (using the hg38 genomic assembly). To further validate the transcript fusion between DAXX and KIFC3 in this sample we isolated RNA from both G292 cells and a telomerase positive osteosarcoma cell line, SJSA1. Following RT-PCR with primer pairs that flanked the predicted fusion sites we confirmed the presence of a single PCR product in the G292 cell line that was not amplified in the SJSA1 control suggesting that a single mature transcript had been generated (Figure 1B). We then purified the PCR product and analyzed the DNA by Sanger sequencing (Figure 1C). The sequencing reaction confirmed a transcript fusion event between exon 7 of DAXX and exon 10 of KIFC3 (Figure 1D).

### Characterizing the structural rearrangement in the DAXX gene locus

**Figure 2:**
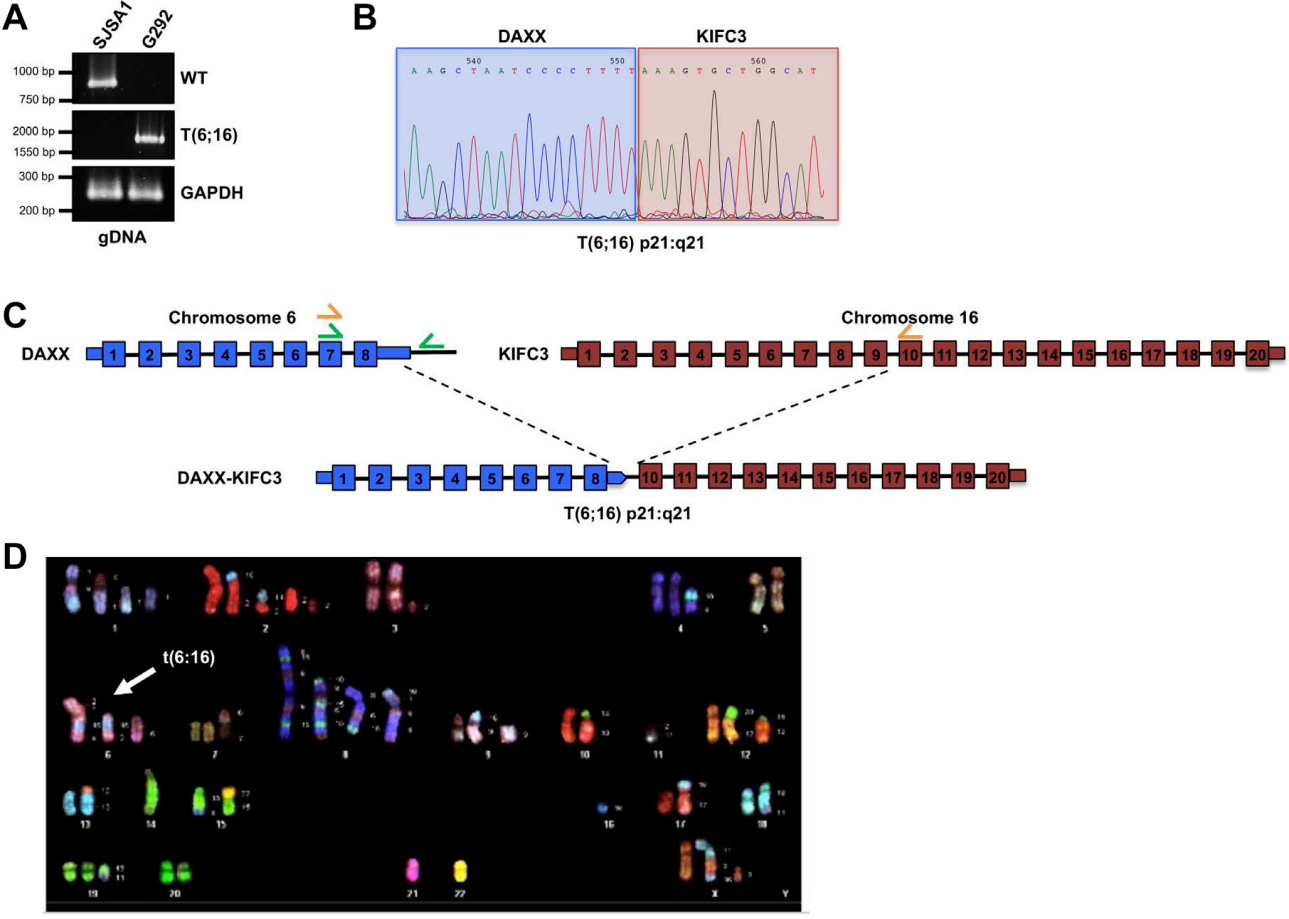
Structural characterization of the DAXX gene locus (**A**) PCR amplification of the wild type DAXX gene locus or the DAXX-KIFC3 fusion using genomic DNA isolated from SJSA1 or G292 cells. GAPDH was amplified as a control. (**B**) Representative chromatogram from Sanger sequencing of the DAXX-KIFC3 gene fusion PCR product amplified from G292 cells. Sequence shown highlights the region where the fusion event occurs at the genomic DNA level now defined as t(6:16). (**C**) Schematic of the t(6;16) fusion in genomic DNA in G292. The 3’UTR of DAXX is fused to intron 9 of KIFC3. Wild type DAXX primers are shown in green, t(6;16) primers are shown in orange. (**D**) Representative metaphase from spectral karyotyping (SKY) analysis of G292 cells.

To further define the genomic rearrangement that gave rise to the DAXX-KIFC3 fusion transcript we analyzed the genomic DNA isolated from G292 and SJSA1 cells. As with our previous analysis on the isolated RNA, we isolated genomic DNA from either SJSA1 or G292 and performed PCR analysis with primer pairs that flanked the predicted fusion sites. While we were unable to detect a PCR product in the genomic DNA from SJSA1 cells, we were able to detect a single band in the genomic DNA from G292 cells (Figure 2A). Following gel extraction and Sanger sequencing of this PCR product we were able to confirm a genetic fusion between DAXX and KIFC3 (Figure 2B). Interestingly, our data demonstrate that the fusion of the two genes lies between the 3′UTR of DAXX and intron 9 of KIFC3 (Figure 1C, 1D). Thus, while the coding region of exon 8 of the DAXX gene is intact following translocation, this exon is spliced from the mRNA transcript. Therefore, in addition to the translocation at the DNA level, there are also defects in DAXX splicing that contribute to the expression of the fused transcript.

Given the importance of DAXX function in heterochromatin integrity, we were also interested in determining whether the G292 cells also maintained a copy of the wild-type DAXX allele. The genomic data demonstrate that the 3′UTR of DAXX is fused to KIFC3 leading to either displacement, or loss, of the genomic DNA downstream of the DAXX gene on the mutant allele. However, if the wild-type allele of DAXX were present in the sample we could detect WT DAXX using primers that recognized the genomic DNA downstream of the DAXX 3′UTR. Therefore, we generated primers that encompassed the genomic DNA on chromosome 6 between exon 7 of DAXX and the intergenic region between DAXX and the 5′UTR of the neighboring gene, ZBTB22 (Figure 2C). While we were able to amplify a PCR product from the genomic DNA isolated from SJSA1 cells, we were unable to generate a PCR product from the genomic DNA of the G292 cells (Figure 2A). Following purification of the PCR product and Sanger sequencing we were able to confirm that the product generated from the genomic DNA of SJSA1 contained the predicted region on chromosome 6 between DAXX and the ZBTB22 gene. Given that we were unable to detect this DAXX-containing product in the G292 cell line these data suggest that the DAXX-KIFC3 fusion mutation is accompanied by loss of heterozygosity for the remaining WT DAXX allele.

To further define the DAXX-KIFC3 translocation event we performed spectral karyotype (SKY) analysis on metaphases spreads prepared from G292 (Figure 2D). This analysis revealed an abnormal human female karyotype with a modal chromosome number of 60. Consistent with osteosarcoma tumors, the G292 sample demonstrated whole chromosome gains and losses and a large number of structural rearrangements including whole chromosome arm deletions and translocations (Supplementary Figure 5). Of the twenty metaphase spreads analyzed 100% demonstrated a clear translocation between chromosome 6 and chromosome 16 with no evidence of unperturbed chromosomes 6 or 16 in this cell line. The translocation, now referred to as t(6:16)(6p21:16q21), was not found in metaphase spreads isolated from normal peripheral blood monocytes grown in culture (Supplementary Figure 6). These analyses confirm that fragments of chromosome 16 containing the KIFC3 gene aberrantly fused to the DAXX gene on chromosome 6. Furthermore, this translocation was found in multiple copies in each cell suggesting genome reduplication following the translocation event that was then selected for during the evolution of the tumor. In addition to this translocation, our data highlight the presence of complex genomic rearrangements of chromosome 8, 6, and 15 that were also present in 100% of the metaphase spreads analyzed indicative of chromoanagenesis (Figure 2D and Supplementary Figure 5) [23].

### Characterizing the function of the DAXX-KIFC3 chimeric protein

**Figure 3:**
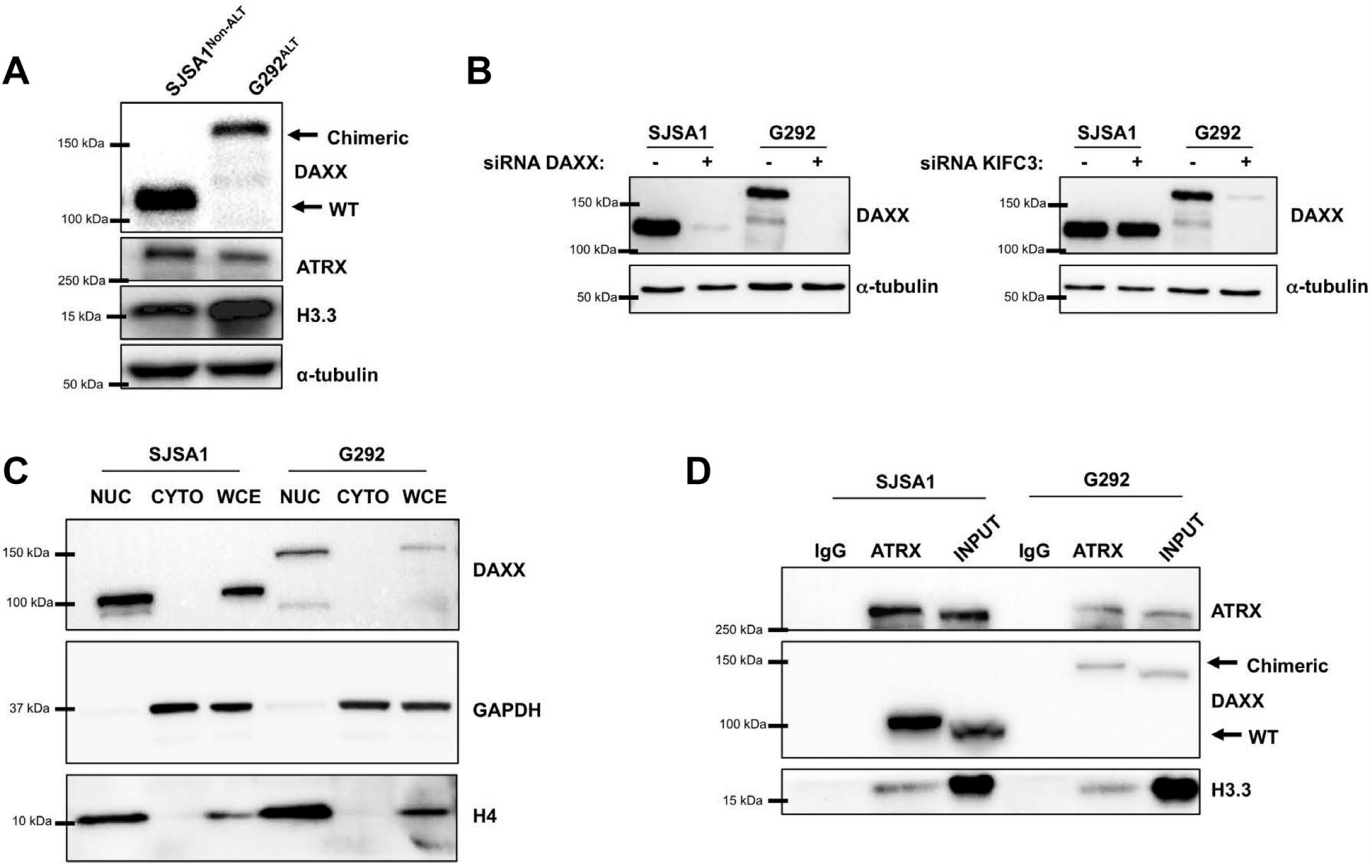
The DAXX-KIFC3 fusion leads to expression of a chimeric protein (**A**) Western blot of SJSA1 and G292 cells for DAXX, ATRX, H3.3 or α-tubulin. Chimeric DAXX-KIFC3 protein runs at a higher molecular weight in G292 cells compared to wild type DAXX in SJSA1 cells. (**B**) Western blot for DAXX in SJSA1 and G292 cells following 48 hour reverse transfection with 100 nM siRNA targeting either DAXX (left panel) or KIFC3 (right panel). α-tubulin was used as a loading control. (**C**) Western blot for DAXX, GAPDH and Histone H4 following cellular fractionation. Nuclear fractions (NUC), cytoplasmic fractions (CYTO) or whole cell extracts (WCE) of SJSA1 and G292. GAPDH and Histone H4 were used to demonstrate successful separation of cytoplasmic and nuclear fractions. (**D**) Immunoprecipitation of ATRX followed by ATRX, DAXX, and H3.3 detection by Western blot in SJSA1 and G292.

Given that the translocation was expressed and the mature transcript preserved the integrity of the fused exons, we asked whether the transcript was also translated into a stable protein product. While SJSA1 cells demonstrated DAXX protein product at the predicted size by western blot, this WT DAXX product was absent in G292 cells. Instead, G292 cells demonstrated a much higher migrating DAXX species, approximately 40 kDa larger than WT DAXX (Figure 3A). To confirm that this higher migrating DAXX species represented a DAXX-KIFC3 fusion protein, we transfected both SJSA1 and G292 cells with siRNA targeting either the N-terminal region of DAXX or the C-terminal region of KIFC3 maintained in the fused transcript. The siRNAs targeting either DAXX or KIFC3, both led to an almost complete loss of the higher migrating DAXX species confirming that this protein product is in fact a DAXX-KIFC3 chimeric protein (Figure 3B). We also observed several weak bands that migrated at a lower molecular weight in the G292 sample. Notably these bands were undetectable after treatment with not just the DAXX siRNA, but also the KIFC3 siRNA in whole-cell extracts confirming that these smaller species were likely degradation products or alternatively processed transcripts of the fusion protein and unlikely to be other isoforms of the wild-type DAXX protein.

DAXX has been described to localize within both the cytoplasmic and nuclear compartments of the cell [24–26]. However, as a member of the kinesin super family, KIFC3 functions as a molecular motor that regulates intracellular transport in the cytoplasm [27, 28]. Structurally, KIFC2 and KIFC3 are the only two known kinesin genes that have the motor domain located in the carboxy terminus and maintain minus end-directed motility along microtubule fibers [29]. Following the fusion with DAXX, the KIFC3 gene retained the microtubule-binding domain. The cytoplasmic localization of KIFC3 and association with microtubules, coupled with the nuclear localization of DAXX led us to speculate that KIFC3 may sequester DAXX in the cytoplasm inhibiting nuclear DAXX function. To further characterize the effects of the DAXX-KIFC3 fusion, we analyzed the cellular localization of the chimeric protein in both SJSA1 and G292 cells. Following cellular fractionation, we detected the majority of the DAXX protein localized within the nuclear compartment in both SJSA1 and G292 cells suggesting that the fusion of DAXX to KIFC3 does not affect DAXX localization within the nucleus.

### Defects in DAXX-KIFC3 function

Our Sanger sequencing results from the RNA fusion transcript demonstrated that the N-terminal portion of DAXX within the chimeric protein retained both the ATRX and H3.3 binding domains. Given that both ATRX and H3.3 proteins were expressed in G292 cells (Figure 3A) we asked whether the ATRX, DAXX, and H3.3 complex was preserved in the presence of the DAXX-KIFC3 fusion protein. Although we would have preferred to immunoprecipitate DAXX directly, several attempts using multiple different DAXX antibodies were unsuccessful. Therefore, we immunoprecipitated ATRX from either SJSA1 or G292 cells and confirmed that the interaction between ATRX, DAXX, and H3.3 was retained in G292 cells (Figure 3D).

**Figure 4:**
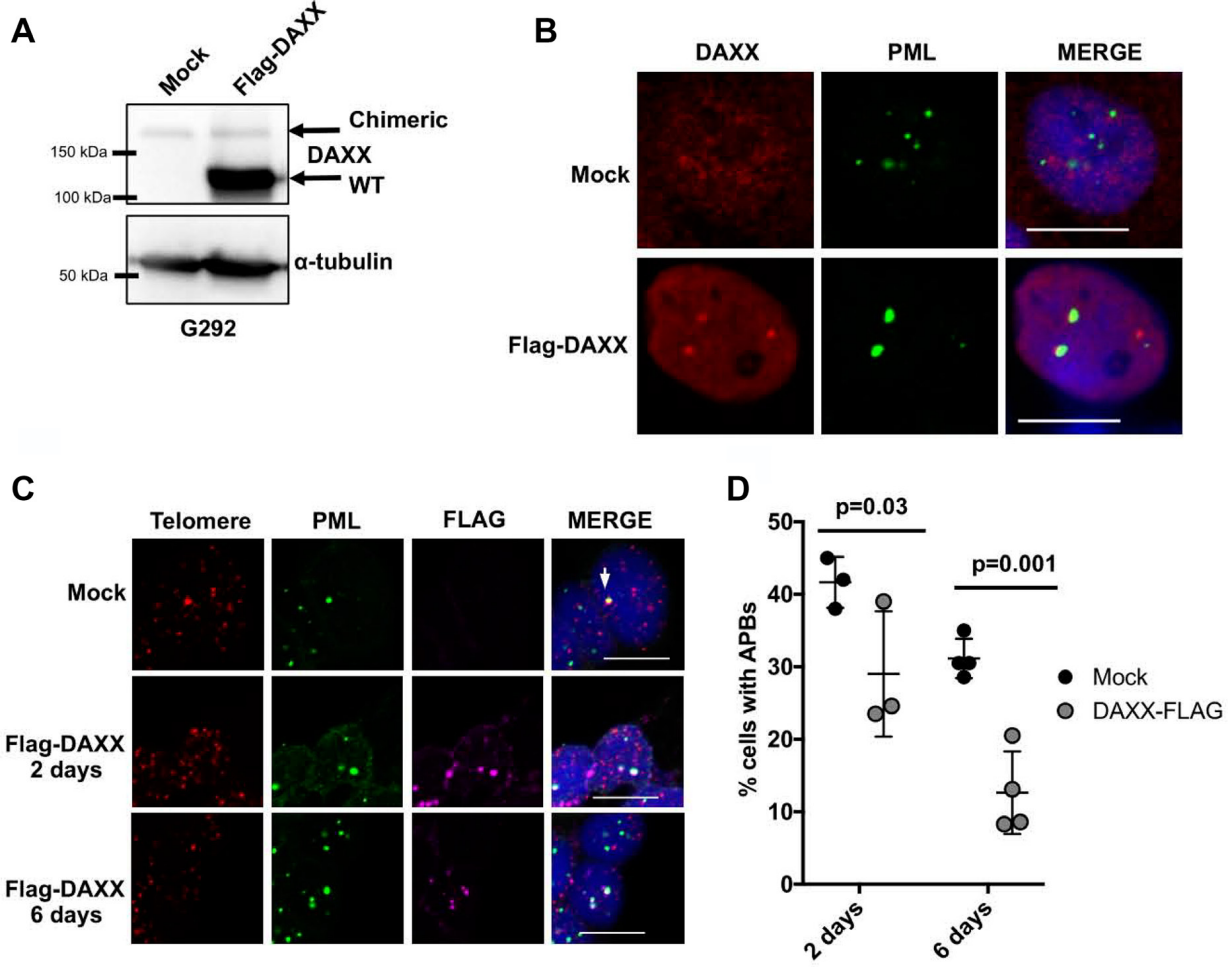
Expression of WT DAXX represses APB formation (**A**) Western blot of G292 cells either mock transfected or transfected with wild-type Flag-DAXX detecting DAXX or α-tubulin. Both chimeric DAXX and Flag-DAXX are detected in the transfected cells, with chimeric DAXX running at a higher molecular weight than wild-type Flag-DAXX. (**B**) Representative images from immunofluorescence analysis of G292 cells mock transfected or G292 cells transfected with Flag-DAXX for 2 days. Cells were stained for DAXX, PML and DAPI. Scale bar is 10 µm. (**C**) Representative images of combined immunofluorescence-FISH analysis of APBs in mock transfected G292 cells or G292 cells transfected with Flag-DAXX for 2 or 6 days. Cells were stained by immunofluorescence for PML and FLAG, and by FISH for telomeres. Arrow indicates APB in a positive cell. Scale bar is 10 µm. (**D**) Quantification of APB data shown in (C), counting a cell positive for APB if it has ≥ 1 large or 3 small telomeres colocalization events and counting a cell positive for Flag-DAXX transfection if it has ≥ 3 Flag foci. *P*-values denoted comparing mock and Flag-DAXX transfected cells at each time point using two-way ANOVA followed by Bonferroni test for multiple comparisons. *n* ≥ 3 independent experiments with ≥ 150 Flag-DAXX positive cells counted total.

DAXX localization and function have also been linked to sumoylation and sumo-mediated interactions [24, 25, 30–32]. While the N-terminus of DAXX is retained after fusion with KIFC3, the extreme C-terminus of DAXX including exon 8, is completely lost. Exon 8 is composed of amino acid residues 733–740 and constitutes a sumo interacting motif (SIM). The DAXX SIM is required not only for recognition of other sumoylated proteins, but also essential for DAXX sumoylation itself [31]. Functionally, the DAXX SIM is essential to mediate an interaction with PML and regulate the association of DAXX to PML nuclear bodies [31, 32]. Moreover, the C-terminus of DAXX has been demonstrated to be essential for the recruitment of DAXX to telomeres [33]. Thus, the DAXX-KIFC3 chimeric protein would not retain wild-type DAXX function either within PML nuclear bodies or at telomeric DNA, loss of both of these activities have been hypothesized to promote ALT activity. To determine whether DAXX-KIFC3 fusion protein localized to PML we used G292 cells alone, or G292 cells that overexpressed the Flag-tagged DAXX and analyzed the localization of DAXX to PML nuclear bodies by immunofluorescence (Figure 4A). As expected, the exogenously expressed DAXX protein forms distinct nuclear foci that colocalize to PML nuclear bodies. However, the endogenous DAXX-KIFC3 fusion protein did not localize to PML confirming defects in DAXX protein function (Figure 4B).

Given that the DAXX-KIFC3 fusion protein fails to fully establish wild-type DAXX function we reasoned that this deficit in DAXX function was contributing to the ALT phenotype. To confirm this, we asked whether expression of exogenous WT DAXX and consequently, partial restoration of DAXX function could repress ALT activity. Previous studies have demonstrated that expression of ATRX in an ATRX deficient ALT positive cancer cell can repress ALT phenotypes including the formation of APB [34]. Therefore, we analyzed APB formation by immunofluorescence in control G292 cells or cells that had been transfected with WT DAXX. While the control cells consistently maintained 35% of cells positive for APB, the cells expressing WT DAXX demonstrated a significant and progressive decrease in APB at both 2 and 6 days following transfection (Figure 4C–4D). Taken together, re-expression of WT DAXX in G292 cells partially restored DAXX function and repressed ALT phenotypes.

## DISCUSSION

Cancer cells can overcome telomere attrition and promote cellular immortality by exploiting mechanisms of telomere elongation. Reactivation of the enzyme telomerase, or activation of the ALT pathway, account for cellular immortalization in the majority of human cancers. Although the ALT mechanism is active in roughly 5% of all human cancers, this incidence skyrockets to approximately 60% in some of the most aggressive forms of human cancer, including osteosarcoma. There is currently an unmet clinical need for the development of novel therapeutic strategies in the treatment of these highly aggressive cancers. However, the mechanistic basis of ALT activation has not been fully elucidated. The identification of genetic mutations in ATRX, DAXX, H3.3, and more recently SMARCAL1 in ALT positive cancers has been instrumental in further defining the molecular basis of ALT. However, defects in ATRX, DAXX, and H3.3 protein function have also been described in the absence of a clear mutation in the coding sequence suggesting alternative mechanisms to functionally inactivate the ATRX/DAXX/H3.3 axis. Our data characterize a translocation between the untranslated region of the DAXX gene and a central intron of the kinesin family member KIFC3 that leads to the expression of a DAXX-KIFC3 fusion protein. The fusion causes defects in DAXX protein function and contributes to activation of the ALT pathway. Interestingly, the cytogenic band on chromosome 6 containing the DAXX gene, 6p21, and the cytogenic band on chromosome 16 containing KIFC3, 16q21 have both been identified as common fragile sites within the genome suggesting that these regions are unstable and may be susceptible to DNA double-strand breaks and ultimately, genomic rearrangements [35–37]. To our knowledge this is the first report to characterize a defect in DAXX protein in osteosarcoma highlighting a novel mechanism for functional inactivation of DAXX in ALT.

Osteosarcoma is rare, with approximately 800 cases reported in the United States each year [38]. Thus, the availability of tumor tissue and or large data sets containing sequence information is limited. To determine the frequency of the t(6:16) translocation in osteosarcoma we looked to analyze existing publicly available datasets for osteosarcoma tumors. The chimeric reads identified in our RNA sequencing analysis would not have been detected by whole-exome sequencing as the coding region of DAXX is wild-type. Therefore, our search was limited to studies that had generated whole genome sequencing or RNA sequencing data. To our knowledge, the Osteosarcoma Genomics study (dbGaP Study Accession pht004384.v1.p1) is one of the largest publicly available datasets for osteosarcoma and this study contains RNA sequencing data for 35 tumors [39] highlighting the limited availability of next generation sequencing in pediatric osteosarcoma. While the DAXX-KIFC3 fusion in G292 was annotated in the CCLE we were unable to detect the t(6:16) translocation in the 35 tumor samples from the Osteosarcoma Genomic Study and this translocation was not annotated in the cBioPortal database. In total, 451 mutations have been described for DAXX in cBioPortal across all of the available tumor types. Notably, this database does not contain data specifically for osteosarcoma tumors. Of the DAXX mutations cataloged, approximately 87.1% are substitution mutations, 12.1% are insertion/deletion mutations, and 0.66% are fusion mutations. Of the substitution mutations, several lie in the C-terminus of DAXX. One in particular, 733* leads to the incorporation of a premature stop codon and a truncated DAXX protein that lacks exon 8 highlighting the significance of exon 8 in DAXX function. The three fusion events that have been identified are products of intrachromosomal rearrangements on chromosome 6 and lead to the fusion of DAXX with other genes located in the 6p21 cytogenic band including UHRF1BP1, TAP2, or RGL2. It is unclear whether these fusions generate chimeric proteins, whether they lead to functional inactivation of DAXX, or whether they retain ALT activity. However, it does suggest that the DAXX gene locus is susceptible to genetic rearrangements and that these rearrangements have the potential to drive cellular immortality through ALT in these cancers.

The localization of the ATRX/DAXX complex to chromatin is critical for histone deposition and heterochromatin formation and defects in this process contribute to the activation and/or maintenance of ALT activity. Here, we have defined a novel translocation in osteosarcoma that leads to defects in DAXX protein function despite maintaining a wild-type DAXX coding region. To date, this fusion event has not been identified in any other cancer suggesting that this is a rare event. The limited availability of osteosarcoma tumor samples and/or publicly available datasets containing next generation sequencing for osteosarcoma does not allow us to draw any significant conclusions on the frequency of this fusion event in this disease. However, given the high level of genome instability and massive structural complexities in osteosarcoma our data argue that these tumors in particular should be analyzed using more comprehensive approaches combining next generation sequencing with immunohistochemistry to improve the detection of ATRX/DAXX pathway inactivation.

## MATERIALS AND METHODS

### Cell lines

Cell lines were submitted for Short Tandem Repeat (STR) analysis and certificates of authentication can be provided upon request. G292, SJSA1 CAL78 and HUO3N1 were cultured in RPMI 1640, 10% FBS, 1% Sodium Pyruvate and 1% Penicillin/Streptomycin. HOS were cultured in Eagle’s Minimum Essential Medium, 10% FBS, 1% Pencillin/Streptomycin. HUO9 and NOS1 were cultured in RPMI 1640 5% FBS, 1% Sodium Pyruvate and 1% Penicillin/Streptomycin. NY and MG63 were cultured in DMEM/F12, 5% FBS, 1% Penicillin/Streptomycin. CAL72 were cultured in DMEM/F12, 10% FBS, 1% Penicillin/Streptomycin. U2OS were cultured in DMEM, 10% FBS, 1% Penicillin/Streptomycin. SAOS2 were cultured in RPMI 1640, 10% FBS, 1% Penicillin/Streptomycin. All cells were maintained at 37°C in a humidified incubator with 5% CO2 except for hFOB. hFOB were cultured in phenol red free DMEM/F12, with 10% FBS, 2.5 mM l-glutamine, 0.3 mg/ml G418. FOB were maintained at 34°C in a humidified incubator with 5% CO2.

### RNA sequencing and alignment

Total RNA was extracted from the three biological replicates of each cell line using Qiagen RNeasy Kit according to manufacturer’s instructions for RNA preparation. Samples were submitted to the BU Microarray and Sequencing Core for library preparation and ribosomal RNA reduction using Kapa RNA HyperPrep kit with Riboerase, and sequenced yielding 2 × 75 bp paired-end read datasets. Read library quality was assessed using fastqc (FastQC 2016) and multiqc packages. Illumina adapters were removed and leading and trailing low-quality bases (below quality 30) were trimmed using Trimmomatic. Reads which were less than 36 bases long after these steps were dropped. The surviving reads were then mapped to the hg38 human reference genome using the STAR aligner with GENCODE v27 annotations. To more accurately quantify the reads mapped to novel splice junctions, 2-pass mapping mode was invoked in STAR. The RNA sequencing data discussed in this publication have been deposited in NCBI’s Gene Expression Omnibus [40] and are accessible through GEO Series accession number GSE118488 (https://www.ncbi.nlm.nih.gov/geo/query/acc.cgi?acc=GSE118488).”

### Fusion detection and visualization

The fused-read junctions with statistics of chimeric alignments were filtered to eliminate chimeras with the mitochondrial genome, keeping only the canonical junctions with repeat length less than 5. Chimeric read alignment from the each of the three replicates were combined, and fusions with fewer than 10 supporting reads were filtered out. Genes at either end of the fusion were identified if any. To visualize the fusion between different chromosomes, a circos plot was made using the package OmicCircos.

### Antibodies, probes and plasmids

The following antibodies and probes were used where indicated. ATRX (Cell Signaling, D1N2E), ATRX (Santa Cruz H-300), DAXX (Cell Signaling 25C12), FLAG-M2 (Sigma F1804), GAPDH (Santa Cruz, 0411), Histone H3.3 (Abcam, EPR17899), Histone H4 (Active Motif, 39269), PML (Santa Cruz H-238), PML (Santa Cruz PG-M3), α-Tubulin (Cell Signaling, 11H10), Purified Rabbit IgG (Bethyl Laboratories P120-101), Alexa Fluor 488 conjugated Donkey Anti-Rabbit IgG (Jackson ImmunoResearch), Cy3 conjugated Donkey anti-Rabbit (Jackson ImmunoResearch), Peroxidase conjugated Goat Anti-Mouse IgG (Jackson ImmunoResearch), Peroxidase conjugated Goat Anti-Rabbit IgG (Jackson ImmunoResearch). PNA Telomere probe (TelC-Cy3, PNA Bio Inc.) Flag-DAXX/pRK5 was a gift from Xiaolu Yang (Addgene plasmid #27974).

### PCR amplification & gel electrophoresis

Cell pellets for each cell line were collected from actively growing cells. Genomic DNA (gDNA) and total cellular RNA were then extracted from the cell pellets using the QIAamp DNA Mini Kit (Qiagen #51304) and RNeasy Mini Kit (Qiagen #74106) protocols according to manufacturer’s instructions. For RT-PCR experiments, 0.5 μg of total cellular RNA was converted to complementary DNA (cDNA) using the SuperScript IV Reverse Transcriptase protocol (Life Technologies #18090010) in a volume of 20 μl according to manufacturer’s instructions. gDNA or cDNA samples underwent three-step PCR amplification using Phusion High-Fidelity PCR Master Mix (Thermo Fisher #F-532S) according to manufacturer’s instructions. PCR reaction parameters were as follows: [1] 0:30 at 98°C; [2] 0:10 at 98°C; [3] 0:30 at annealing temperature (60°C for GAPDH primers, 65°C for DAXX related primers); [4] 0:10 at 72°C; [5] Repeat steps 2–4 for 29 cycles; [6] 10:00 at 72°C [7] Hold at 12°C. PCR products were then resolved using agarose gel electrophoresis, stained using SYBR Gold (Thermo Fisher #S-11494), and visualized using a BioRad ChemiDoc XRS+ imaging system.

Primers: *DAXX Exon 7 Forward* [5′: GTG GAA AGG CAA AGG TCA GT: 3′]; *DAXX-ZBTB22 Intergenic Reverse* [5′: GAG GCA TTA TCG CTT GAG ACT G: 3′]; *KIFC3 Exon 9 Reverse* [5′: GAG CTC ATT GTG GCA CTT CTT A: 3′]; *GAPDH Forward* [5′: CAG AAC ATC ATC CCT GCC TCT AC: 3′]; *GAPDH Reverse* [5′: TTG AAG TCA GAG GAG ACC ACC TG: 3′].

### Sanger sequencing

Following visualization by gel electrophoresis, DNA was purified from individual bands using the GeneJET Gel Extraction Kit (Thermo Fisher #K-0691) according to manufacturer’s instructions. Samples were submitted for Sanger Sequencing to Genewiz, Inc. (Cambridge, MA, USA) premixed in a volume of 15 μl containing 20 ng purified PCR product mixed with 25 pmoles primer templates. The previously described DAXX Exon 7 Forward, DAXX-ZBTB22 Intergenic Reverse, and KIFC3 Exon 9 Reverse primers sequences were used for sequencing.

### Spectral karyotyping

Spectral Karyotype analysis was performed at Roswell Park Cancer Institute Pathology Resource Network. Cells were treated overnight with 0.03 ug/ml of colcemid then harvested and fixed. Metaphase spreads from fixed cells were then hybridized with SKY probe (Applied Spectral Imaging) for 36 hours at 37 degrees Celsius. Slides were then prepared for imaging using ASI’s CAD antibody kit and counterstained with DAPI. Twenty metaphase spreads were then captured and analyzed using ASI’s HiSKY software.

### Western blot

Western blots were performed using standard protocols. Briefly, cells were collected by trypsinization and washed with ice-cold 1XPBS. Samples were then lysed in 2X Sample Buffer at 95°C for 15 minutes before being sonicated in a water bath at 4°C for 10 minutes (20-second pulse on/30-second pulse off at 100% amplitude). Soluble protein lysates were then analyzed by western blot using standard SDS-PAGE techniques and transferred onto PVDF membranes. Membranes were blocked in TBST (1X TBS, 0.1% Tween-20) containing 5% milk and then incubated overnight at 4°C with the appropriate primary antibodies. Following overnight incubation with primary antibodies, membranes were washed 3 times in TBST, then incubated with peroxidase conjugated secondary antibodies and visualized using enhanced chemiluminescence reagents from BioRad and Thermo Fisher.

### siRNA reverse transfection

For reverse transfection experiments, SJSA1 and G292 cells were initially seeded at a density of 150,000 cells/well and 220,000 cells/well respectively in a 6-well plate and either mock transfected or transfected with siRNA SMARTpool (GE Darmacon). Cells were transfect with 100 nM pooled siRNA using Lipofectamine RNAiMax (Thermo Fisher #18324012) diluted in Opti-MEM according to manufacturer’s instructions. 24 hours after initial transfection, the siRNA solution was removed and replaced with fresh media. Whole cell lysates were collected for Western blot 48 hours after reverse transfection.

siRNA Targeting Sequences: *DAXX Pool LQ-004420-00-0002* → J-004420-05 (CAG CCA AGC UCU AUG UCU A); J-004420-06 (GAG GUU AAC AGG CGC AUU G); J-004420-07 (GCS AAA CAA AGG ACG CAU A); J-004420-08 (GGA GUU GGA UCU CUC AGA A); *KIFC3 Pool LQ-008338-00-0002* → J-008338-07 (GCU CUA UUC CCU CAA GUU U); J-008338-08 (CCA AUG CUG UGA CUU UCG A); J-008338-09 (GGU CAA GCC AGG AGC AUC U).

### Cellular fractionation

Cells were collected by trypsinization and lysed in CSK buffer (10 mM Hepes pH 7.5, 10 mM KCl, 2 mM MgCl_2_, 300 mM sucrose, 10% glycerol, 0.1% Triton-X-100) for 5 minutes on ice and then centrifuged for 5 minutes at 1,400 × g at 4°C. The supernatant was collected and analyzed as the cytoplasmic fraction. The remaining cell pellet was again lysed in CSK buffer with 0.25% Triton-X-100 for 5 minutes on ice, and centrifuged for 5 minutes at 1,400 × g at 4°C. The supernatant was discarded and the cell pellet was washed in CSK buffer without Triton-X-100 and centrifuged for 5 minutes at 1,400 × g at 4°C. The remaining cell pellet was then resuspended in NETN lysis buffer (150 mM NaCl, 20 mM TRIS-HCl pH 8.0, 0,5 mM EDTA, 0.5% NP-40) for 30 minutes on ice with intermittent agitation. Samples were sonicated for a total of 10 minutes at 4°C (20-second pulse on/30-second pulse off at 100% amplitude) and centrifuged for 10 minutes at 4 degrees at 17,000 × g. The supernatant was collected and analyzed as the nuclear fraction.

### Immunoprecipitation

Cells were lysed in NETN buffer (150 mM NaCl, 20 mM TRIS-HCl pH 8.0, 0,5 mM EDTA, 0.5% NP-40) on ice for 30 minutes with intermittent agitation followed by sonication (QSonica Q800R3) for a total of 10 minutes at 4°C (20-second pulse on/30-second pulse off at 100% amplitude). Samples were then centrifuged at 4°C for 10 minutes at 17,000 × g. The supernatant was moved to a fresh Eppendorf tube and a portion of the sample was removed to use as the input. 3 ug of antibody (ATRX, Cell Signaling Technology D1N2E; PML, Santa Cruz Biotechnology PG-M3; IgG, Bethyl Laboratories) was added to the remainder of each sample and incubated overnight at 4°C with gentle shaking. The following day the samples were incubated with Protein A (ATRX) or Protein G (PML) coated magnetic beads (Thermo Fisher Scientific 10001D, 10003D) for 30 minutes at room temperature. Beads were collected using magnetic separation, and subsequently washed in NETN buffer twice for 5 minutes each. Finally, beads were resuspended in 2X SDS sample buffer and boiled for 5 minutes before analysis by SDS PAGE.

### Flag-DAXX overexpression

G292 cells were forward transfected with Flag-DAXX/pRK5 using Fugene 6 transfection reagent (Promega). Cells were seeded onto coverslips overnight. The following day, 1.5 µl of Fugene 6 transfection reagent was mixed with Opti-MEM, and 0.5 µg of Flag-DAXX/pRK5 in a total volume of 50 µl. After 15 minutes, this mixture was added into a total volume of 500 µl of media in a 24 well dish. Transfection mixture was removed and media was replaced after 6–16 hours. For combined immunofluorescence and DNA FISH experiments, cells were reseeded after 48 hours and transfection was repeated after 72 hours. Overexpression was confirmed using western blot for DAXX (Cell Signaling 25C12).

### Immunofluorescence

For immunofluorescence, cells were rinsed with PBS and then pre-extracted with 0.25% Triton-X in PBS for 1 minute at room temperature. Following pre-extraction, cells were rinsed with PBS, and then fixed in 4% paraformaldehyde for 10 minutes at room temperature. Cells were rinsed with PBS and then permeabilized with 0.5% Triton-X in PBS for 15 minutes at room temperature. Cells were incubated in blocking buffer (3% BSA, 0.05% Tween-20 in PBS) for 10 minutes, and then incubated in primary antibody in blocking buffer overnight at 4°C (PML Santa Cruz PG-M3 1:300; DAXX Cell Signaling 25C12 1:50). The next day, following three 5 minute washed in PBS, cells were incubated in secondary antibody (anti-Rab Cy3 1:250; anti-Mouse AlexaFluor 488 1:250) diluted in blocking buffer for 1 hour at room temperature. Finally, cells were washed three times for 5 minutes in PBS at room temperature, with DAPI added into the final wash. The coverslips were mounted on glass microscope slides with Vectashield mounting medium and analyzed using a Zeiss LSM 710 confocal microscope.

### Combined immunofluorescence and DNA fluorescence *in situ* hybridization

For combined immunofluorescence and DNA FISH, cells were rinsed with PBS and then treated with cytobuffer (100 mM NaCl, 300 mM Sucrose, 3 mM MgCl_2_, 10 mM PIPES pH 7, 0.1% Triton X-100) for 7 minutes at 4°C. Cells were then rinsed with PBS and fixed in 4% paraformaldehyde for 10 minutes at room temperature. Cells were permeabilized in 0.5% NP-40/PBS for 10 minutes and then blocked in PBG (0.5% BSA, 0.2% fish gelatin, PBS) for 1 hour at room temperature. Cells were incubated in PML antibody (Santa Cruz H-238, 1:500) diluted in PGB overnight at 4°C. Cells were washed three times with PBS for 5 minutes each and subsequently incubated with secondary antibody (Jackson ImmunoResearch Alexa Fluor488 conjugated donkey anti-Rabbit, 1:250) diluted in PBG for 45 minutes at room temperature. The cells were washed three times with PBS for 5 minute each, then fixed with 4% paraformaldehyde for 10 minutes at room temperature. Cells were digested with RNaseA 200 µg/mL diluted in 2X SSC for 30 minutes at 37°C. Cells were dehydrated in a series of ethanol washes, 70%, 85% then 100% for 2 minutes each at room temperature. The coverslips were dried at 37°C for 20 minutes. 10 nM telomere probe (PNA-Bio Tel-Cy3) was added to coverslips in hybridization buffer. Slides were denatured at 85°C for 3 minutes, and then placed in a humidified chamber overnight at 37°C. The following day, coverslips were washed in 2X SSC + formamide (mixed 1:1), three times for 5 minutes each at 37°C, then washed in 2X SSC three times for 5 minutes each at 37°C, and finally one time in 2X SSC containing DAPI (4′, 6-diamidino-2-phenylinodole, dihydrochloride) for 20 minutes at room temperature. The coverslips were mounted on glass microscope slides with Vectashield mounting medium and analyzed using a Zeiss LSM 710 confocal microscope.

## SUPPLEMENTARY MATERIALS FIGURES


