# Supplementary material for: Identification of a novel gene fusion in ALT positive osteosarcoma
Source: Oncotarget. 2018 Aug 28;9(67):32868–80. doi: 10.18632/oncotarget.26029 (PMC6132345; doi:10.18632/oncotarget.26029)
Supplement: Supplementary file 1 [file oncotarget-09-32868-s001.pdf]

Identification of a novel gene fusion in ALT positive osteosarcoma

SUPPLEMENTARY MATERIALS

Antibodies

The following antibodies were used where indicated. PCNA (Cell Signaling D3H8P), SMARCAL1 (Santa Cruz, sc-376377).

Primers

ATRX Exon 1 Forward – 5′ – AATGGCTGACGG AAAGAGAAAGAG – 3′
ATRX Exon 1 Reverse – 5′ – CTCATGGGCTCA GCGGTCATG – 3′
ATRX Exon 9 Forward – 5′ – GTGGTGTGCGGA AGGTGGAAAC – 3′
ATRX Exon 9 Reverse – 5′ – GGAGTTCATGT TGGCTGTGGTC – 3′
ATRX Exon 35 Forward – 5′ – GGAAATTTCACG ACTAGAATGTCC – 3′
ATRX Exon 35 Reverse – 5′ – CTTTGGGACTGG CTCAGATTATAG – 3′

C-circle assay

The C-circle assay was performed as previously described [1]. Briefly, genomic DNA was isolated using the Qiagen QiaAMP DNA Mini Kit according to the manufacturer’s instructions. Purified DNA was digested with AluI and MboI restriction enzymes at 37°C overnight. Digested DNA was purified using a Qiagen PCR clean-up kit according to the manufacturer’s instructions. The DNA was quantified with a Nanodrop spectrophotometer and then diluted to 10 ng/µl. 40 ng of gDNA was diluted in 25 µl of 1X Φ29 buffer (NEB) containing BSA (NEB; 0.08 mg/ml), 0.1% Tween, 0.25 mM each dATP, dGTP, dTTP and incubated in the presence or absence of 7.5 U Φ29 polymerase (NEB) at 30°C for 8 hours, following by 65°C for 20 minutes.

Amplification products were diluted to 10X SSC and run through a dot blot apparatus onto a Hybond N+ membrande using a BioRad dot blot vacuum manifold. The membrane was crosslinked for 35 s (125J). The membrane was incubated in Ultra-Hyb hybridization buffer (Ambion) for 1 hour at 50°C. Telomeric probe $(CCCTAA)_4$ was labeled using the DIG oligonucleotide 3′- end labeling kit (2nd generation, Roche) according to manufacturer’s instructions. DIG labeled probe was added to the Ultra-Hyb hybridization buffer (1:1000) and incubated overnight at 50°C. The following day, the membrane was washed twice with 2X SSC + 0.1% SDS at room temperature for 5 minutes each and twice with 0.5X SSC + 0.1% SDS at 50°C for 15 minutes each. The membrane was developed using anti-DIG-AP (Roche), CDP-star (Roche), and the DIG Wash and Block Buffer set (Roche) following manufacturer’s instructions.

Combined immunofluorescence for PCNA and DNA fluorescence *in situ* hybridization

For PCNA staining combined with telomere FISH, the same combined immunofluorescne and DNA FISH protocol used to stain PML and telomeres was followed with one exception. For staining with PCNA, cells were permeabilized in 100% cold methanol at −20°C for 10 minutes instead of 0.5% NP-40. Following permeabilization cells were blocked in PBG (0.5% BSA, 0.2% fish gelatin, PBS) for 1 hour at room temperature.

REFERENCES

1. Henson JD, Cao Y, Huschtscha LI, Chang AC, Au AY, Pickett HA, Reddel RR. DNA C-circles are specific and quantifiable markers of alternative-lengthening-of-telomeres activity. Nat Biotechnol. 2009; 27:1181–85. https://doi.org/10.1038/nbt.1587.

Cell Line	ATRX	DAXX	H3.3	SMARCAL1	ALT STATUS
MG63	✓	✓	✓	✓	Non-ALT
SJSA1	✓	✓	✓	✓	Non-ALT
hFOB1.19	✓	✓	✓	✓	Non-ALT[8]
HOS	✓	✓	✓	✓	Non-ALT
HUO3N1	✓	✓	✓	✓	Non-ALT[8]
HUO9	Abnormal expression<br>No Full Length protein[1]	✓	✓	✓	ALT
NOS1	Exon Deletion[2]<br>No Full Length protein	✓	✓	✓	ALT
NY	✓	✓	✓	No Full Length protein[6]	ALT
CAL72	Exon Deletion[2]<br>No Full Length protein	✓	✓	✓	ALT
CAL78	✓	✓	✓	Deletion[6,7]<br>No Full Length protein[1]	ALT
SAOS2	Abnormal expression<br>No Full Length protein[1,3]	✓	✓	✓	ALT
U2OS	Exon Deletion[2,3,4]<br>No Full Length protein[1]	✓	✓	✓	ALT
G292	✓	T(6;16)[5]	✓	✓	ALT

[1] See Supplementary Figure 3A-B
[2] See Supplementary Figure 3C
[3] As reported in Lovejoy et al PLoS Genetics (2014)
[4] As reported in Heaphy et al Science (2011)
[5] As reported in this manuscript
[6] See Supplementary Figure 3D
[7] As reported in Diplas et al. Nature Communications (2018)
[8] See Supplementary Figure 2A-B

Supplementary Figure 1: Table of all cell lines sequenced with ALT status indicated. ATRX, DAXX, H3.3 and SMARCAL1 status are indicated based on this and previously published data. Check mark indicates that gene is expressed based on RNA Sequencing data.

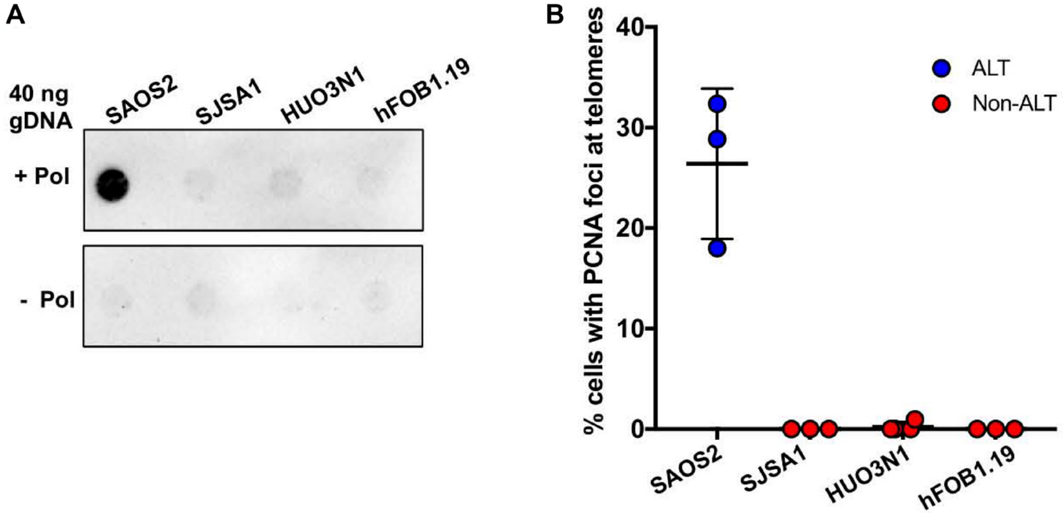

Supplementary Figure 2: (**A**) Dot blot result from C-circle assay performed using 40 ng genomic DNA isolated from SAOS2 (positive control), SJSA1 (negative control), HUO3N1 or hFOB1.19 cells. C-circle assay was performed with or without Phi29 polymerase. (**B**) Quantification of focal PCNA colocalization with telomeric DNA in SaOS2, SJSA1, HUO3N1 or hFOB1.19 cells. Cells were stained for PCNA using immunofluorescence and telomeres using fluorescence *in situ* hybridization with a telomere specific probe. $n = 3$ independent experiments with ≥ 100 cells counted per experiment.

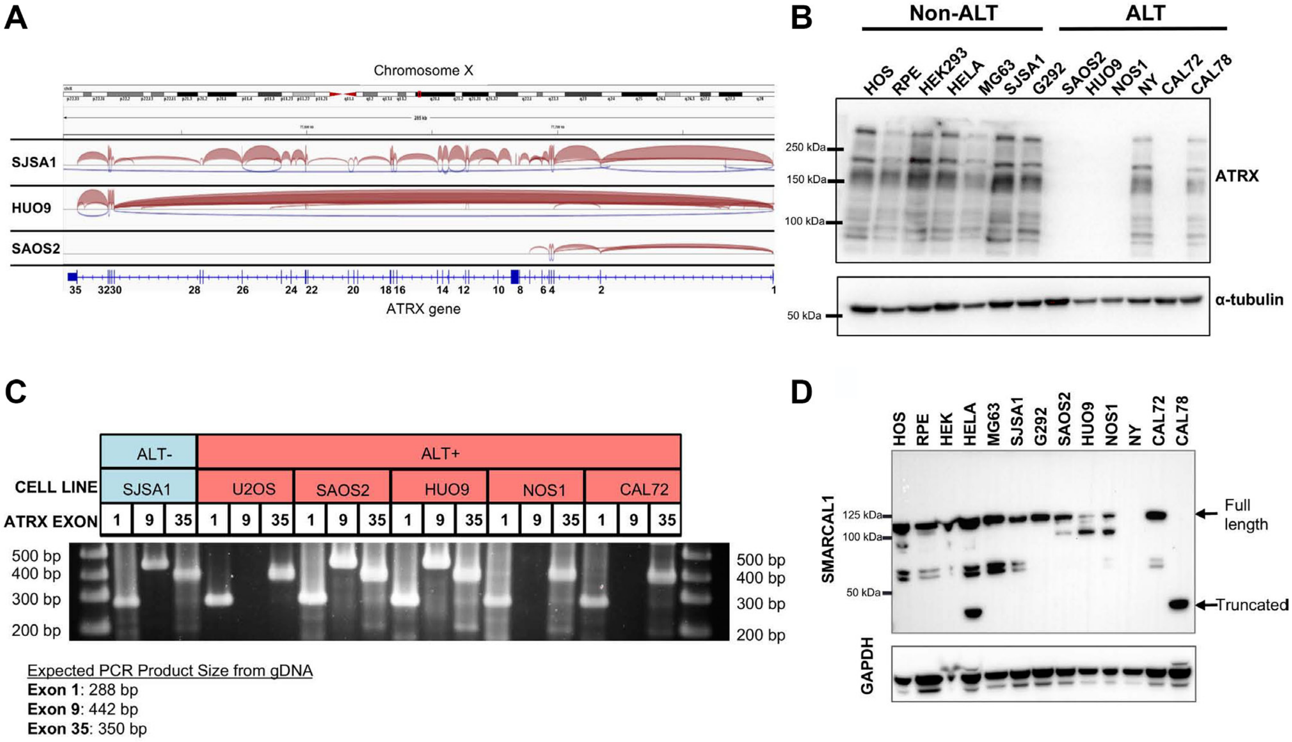

Supplementary Figure 3: (**A**) Representative image from the Integrated Genomics Viewer (IGV) displaying RNA sequencing reads aligned to the ATRX gene (hg38) from SJSA1, HUO9 and SAOS2 cells. Red connecting arcs represent predicted splice junctions between exons in the RNA from each individual cell line. Blue vertical bars in the ATRX reference gene represent the exons. (**B**) Western blot of a panel of cell lines for ATRX. ATRX runs above 250 kDa, the remaining bands represent alternative isoforms or degradation products. (**C**) PCR amplification products of exon 1, exon 9 or exon 35 of ATRX from genomic DNA isolated from SJSA1, U2OS, SAOS2, HUO9, NOS1 or CAL72 cells. (**D**) Western blot on a panel of cell lines using SMARCAL1 or GAPDH antibodies as indicated.

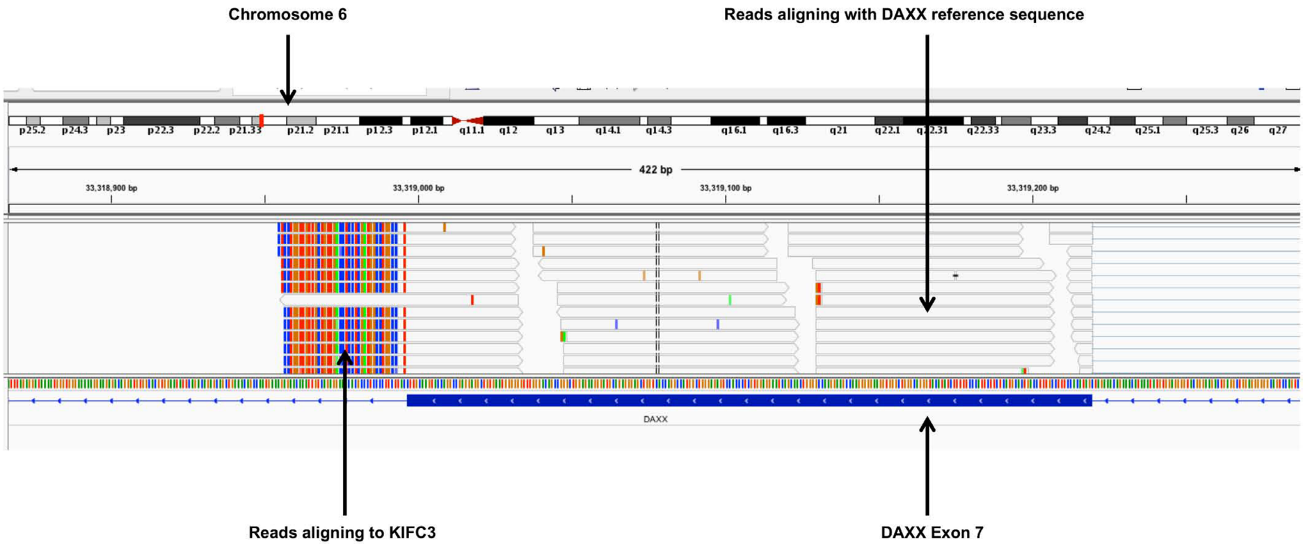

Supplementary Figure 4: Representative image from IGV genome browser displaying RNA sequencing reads aligned to DAXX exon 7 from G292 cells. White bars represent the portion of the sequencing reads that align to DAXX using the reference genome (hg38). Colored bars represent the portion of the read that does not align to DAXX using the reference genome, but instead aligns to the KIFC3 gene (not pictured).

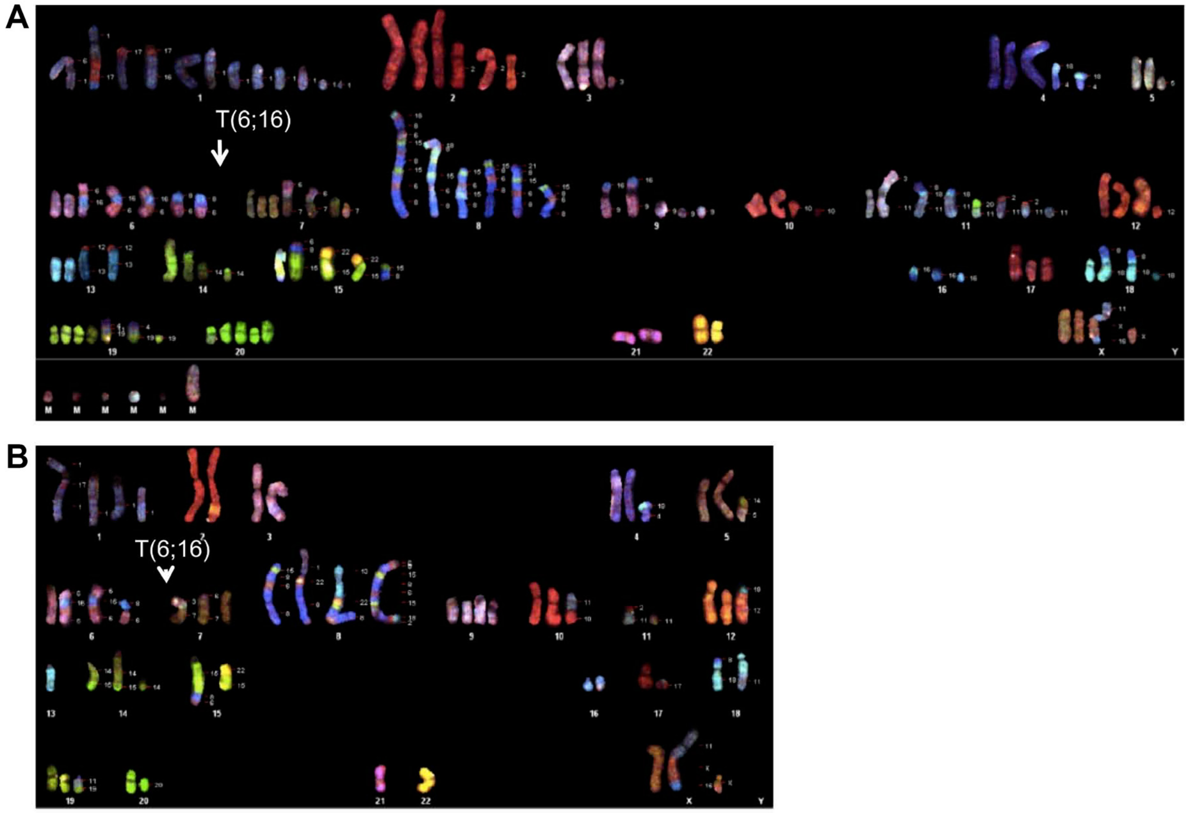

Supplementary Figure 5: Representative G292 metaphase spreads analyzed by spectral karyotyping. The t(6;16) translocation, changes in ploidy, and chromoanagenesis are evident in both (**A** and **B**).

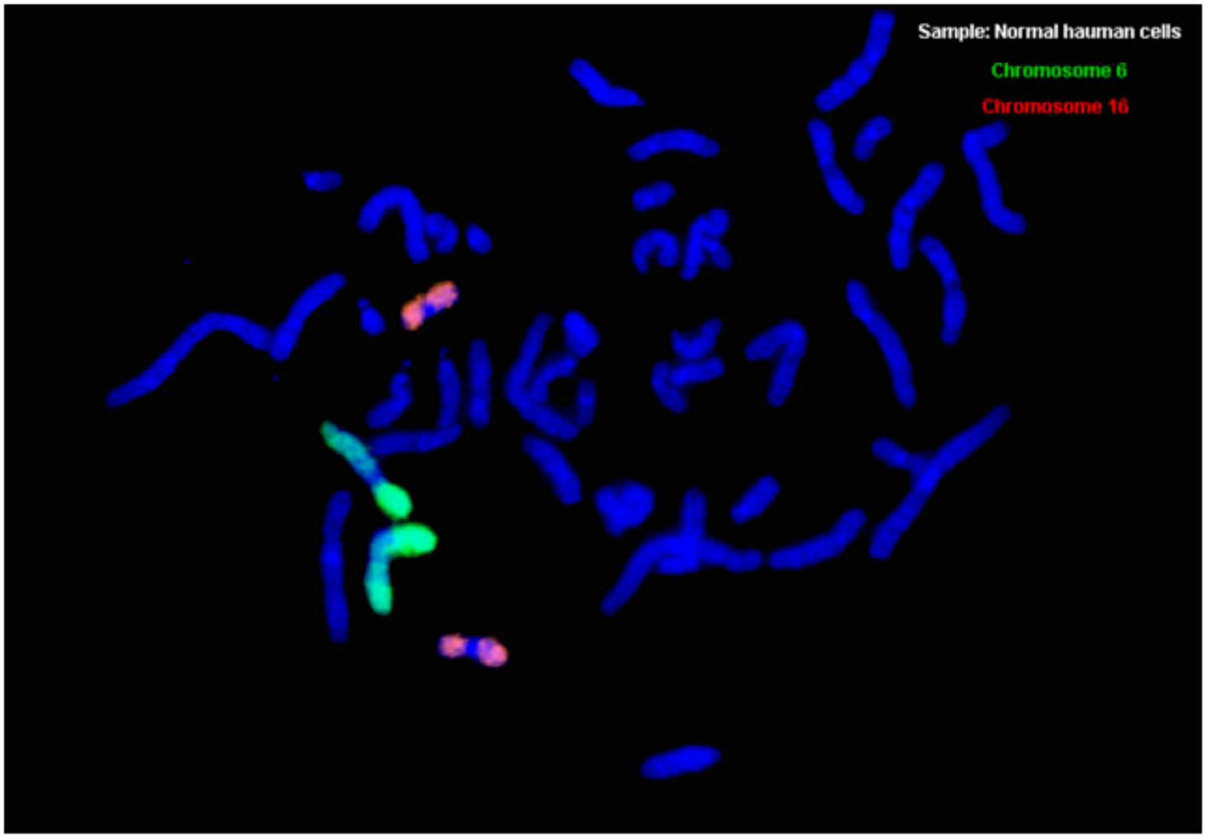

Supplementary Figure 6: Representative metaphase spread prepared from peripheral blood monocytes following FISH with whole chromosome paint for chromosome 6 (green) and chromosome 16 (red). Normal peripheral blood monocytes contain two copies of chromosome 6 and 2 copies of chromosome 16 as expected. Analysis was performed by Brigham and Women's Hospital Department of Pathology CytoGenomics Core.
